# Role of cold atmospheric plasma alone or combined with conventional surface treatments on shear bond strength of 3Y-TZP and 5YSZ ceramics bonded to dentin

**DOI:** 10.2340/biid.v13.45563

**Published:** 2026-05-05

**Authors:** Ahmad Abdulkareem Alnazzawi, Mohamed F. Aldamaty, Mohammed H. AbdElaziz, Ahmed Yaseen Alqutaibi, Ahmed E. Farghal, Jamal Qernas Almarashi, Abdel-Aleam H. Mohamed, Muhammad Sohail Zafar

**Affiliations:** a Department of Substitutive Dental Science, College of Dentistry, Taibah University, Madinah, Saudi Arabia; b Department of Fixed Prosthodontics, Faculty of Dental Medicine, Al-Azhar University, Cairo, Egypt; c Department of Restorative and Aesthetic Dentistry, College of Dentistry, Almaaqal University, Basrah, Iraq; d Department of Physics, College of Science, Taibah University, Madinah, Saudi Arabia; e Department of Clinical Sciences, College of Dentistry, Ajman University, Ajman, United Arab Emirates; f Center of Medical and Bio-allied Health Sciences Research, Ajman University, Ajman, United Arab Emirates; g School of Dentistry, University of Jordan, Amman, Jordan

**Keywords:** Adhesive performance, nonthermal plasma, yttria-stabilized zirconia, translucent zirconia, resin–dentin adhesion

## Abstract

This study evaluated the impact of different surface treatments – cold atmospheric plasma (CAP), airborne particle abrasion (APA), hydrofluoric acid (HF), and their combinations – on the bond strength of two zirconia types (3Y-TZP, 5YSZ) to dentin using self-adhesive resin cement, with or without primer. Two hundred zirconia and dentin specimens underwent surface treatment, cementation, thermocyclic aging, and shear bond strength (SBS) testing. Results showed APA and CAP significantly enhanced SBS (*P* < 0.001), while HF had no effect. Combined treatments (APA+CAP, CAP+HF) slightly reduced SBS. Primer application improved SBS across all groups. APA or CAP with a 10-MDP primer achieved the highest SBS values. CAP is a viable alternative to APA, though APA remains cost effective. HF is not recommended for surface treatment of zirconia. CAP exhibited a slightly higher, yet statistically comparable, SBS between zirconia and dentin when compared with APA. Nonetheless, APA may still represent the simplest and most cost-effective technique available to date. The combination of APA and CAP might not be recommended, as it showed a slight reduction in SBS of the tested specimens and inefficiency in terms of time and energy. Hydrofluoric acid showed no impact on SBS when combined with CAP. Both 3Y-TZP and 5YSZ can provide adequate clinical bond strength with the proper surface treatment and adhesive protocol.

## Introduction

In the last two decades, dental rehabilitation has greatly shifted toward metal-free restorations and prostheses. Zirconia of the oxide ceramic family is one of the most applied materials in dentistry, owing to its good esthetic appeal, biocompatibility, and very good mechanical properties [1, 2]. The first and second zirconia generations are 3mol% yttria-stabilized tetragonal zirconia polycrystalline ceramic (3Y-TZP), a relatively opaque material that is applied mostly as a core structure [3]. To improve the esthetic appeal, yttria contents were increased to 4- and 5-mol%, forming more esthetic two monolithic zirconia restorations, 4mole% yttria-stabilized zirconia ceramic (4YSZ) and 5mol% yttria-stabilized zirconia ceramic 5YSZ [3]. These two material types enable the monolithic use of zirconia in esthetic dental applications, such as full crowns and laminate veneers, particularly in the case of 5YSZ [4, 5]. 4YSZ and 5YSZ contain a significantly high rate of cubic phase over the appreciable tetragonal crystalline phase, resulting in losing the transformation toughening [2] and reducing their fracture toughness than 3Y-TZP [6].

Generally, teeth-supported restorations necessitate teeth preparations, which often involve dentin. It is crucial to have a reliable and durable bond between dentin and zirconia superstructure, particularly in cases where enamel is scarce or depleted, to guarantee the long-term survival of the inserted restorations [7–9]. It is well known that adhesion to enamel is superior to that of partially or fully exposed dentin [10]. To achieve good adhesion between the zirconia superstructure and dentin, it is crucial to follow an optimum adhesion protocol both for dentin and zirconia.

A proper zirconia surface treatment is required for successful adhesion. Over the past decades, airborne particle abrasion (APA) has been the preferred surface treatment protocol, resulting in a very good, reliable, and durable bond, both in laboratory [11–14] and clinical studies [14, 15]. Unlike glass ceramics, which are treated by hydrofluoric (HF) acid etching [16]. Zirconia surface is chemically inert and usually treated by mechanical roughening methods [17]. Nevertheless, APA can result in a damaging impact on zirconia microstructure, such as microcracks, and premature mechanical aging, due to early transformation of tetragonal to monoclinic phase [12], increasing the possibility of microcracks initiation [18, 19]. Therefore, less damaging surface treatment methods have been evaluated in many laboratory studies such as high-temperature HF solution etching [20–22] and zirconia primer applications [11, 23, 24]. Phosphate monomer containing primers and adhesive, such as 10-methacryloyloxydecyl dihydrogen phosphate (10-MDP), showed improved bond strength to zirconia, which has been identified as a chemical approach to enhance the adhesion mechanism of Y-TZP materials [25, 26].

Plasma, recognized as the fourth state of matter, consists of atoms, molecules, and highly energized radicals. This treatment method encompasses both thermal and nonthermal types, with cold plasma operating at near-room temperature and atmospheric pressure [27]. Atmospheric plasma is utilized to activate surfaces, thereby modifying solely the outermost atomic layers of materials. Untreated surfaces typically exhibit a limited number of polar groups available for liquid interaction; in contrast, plasma treatment facilitates the introduction of these polar groups onto the surface molecules [27, 28]. Cold atmospheric plasma (CAP) has been demonstrated to enhance the wettability of ceramic surfaces, potentially leading to improved adhesive penetration and significantly increasing shear bond strength (SBS) [29–31]. Previous studies [30, 31] have primarily focused on zirconia-composite resin bonding, while evidence regarding zirconia bonding to dentin is scarce. Given the clinical relevance of dentin bonding in tooth-supported zirconia restorations, there is a need to evaluate the effect of CAP treatment on zirconia-dentin adhesion.

Therefore, this study investigated the impact of various zirconia surface treatments (CAP, APA, and HF acid) on SBS of two zirconia materials to dentin using self-adhesive resin cement with or without zirconia primer. Three null hypotheses were assigned for this study: (1) Different surface treatments of zirconia would show no impact on SBS, (2) various adhesive cementation approaches with or without zirconia primer would show comparable shear bond strength to dentin, and (3) various types of zirconia may show comparable SBS to dentin.

## Materials and methods

### Study design

This study was conducted at Taibah University after obtaining the ethical approval (Ref No: TUCDREC/210323). The sample size was determined to investigate the effects of four various surface treatments compared to a control group. Each protocol was investigated with or without a primer for two zirconia materials. Sample size was calculated based on the methodologies delineated by Yang et al. [32] and employing the G*Power analysis software (version 3.1.9.4). Accordingly, 180 specimens (9 per subgroup) were adequate for achieving the actual power (1-β error) of 0.8 (80%), identifying a large effect size (f) of 0.51. For a two-sided hypothesis test, a significance level (α error) was 0.05 (5%). To further enhance the statistical power of the study, 200 zirconia specimens with varying yttria content were prepared: 100 3Y-TZP specimens (ceraMotion^®^ Z HT Multishade, Dentaurum, Germany) and 100 5YSZ specimens (ceraMotion^®^ Z Cubic Multishade), which were further subdivided into 20 specimens per subgroup based on five surface treatment applied and subsequently categorized according to the presence or absence of primer (*n* = 10).

### Preparation of zirconia specimens

**Figure 1:**
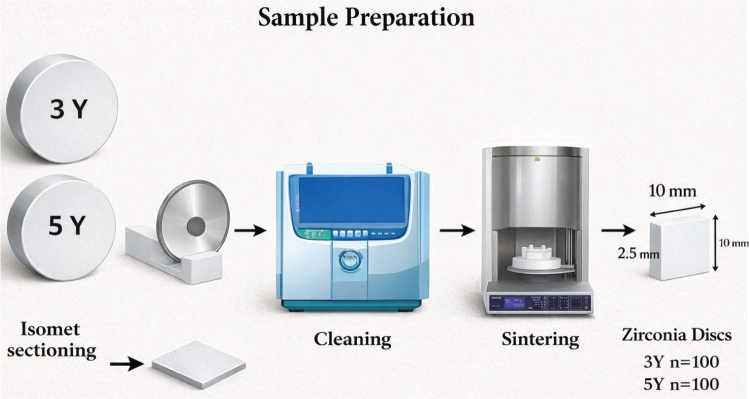
Diagram representing zirconia specimens’ preparation.

Zirconia blanks were sectioned into square-shaped specimens using a precision cutter (IsoMet 4000 microsaw, Buehler). Applying a 0.6-mm diamond disc at a speed of 2,500 rpm with a fixed feed rate (10 mm/min). This was followed by wet-grinding with 600-grit silicon carbide on an Automet 500 (Buehler) for 60s, then cleaning in an ultrasonic bath [33]. According to manufacturer’s instructions, sintering of specimens was done in HTC speed furnace (sintering furnace, Sirona, Germany), resulting in final measurements of 10×10×2.5 mm. The tested samples were cleaned for 3 min using 99% isopropanol in the ultrasonic bath (Figure 1). Table 1 represents details of all materials.

**Table 1:** Materials used in this study.

Materials	Brand	Manufacturer
ZrO_2_ 3Y-TZP (3mol%)	ceraMotion^®^ Z HT Multishade	Dentaurum-Germany
ZrO_2_ 5YSZ(5mol%)	ceraMotion^®^ Z Cubic Multishade	Dentaurum-Germany
Hydrofluoric acid	Porcelain Etch Gel 9.5%	Bisco (USA)
Zirconia primer	Z- prime plus	Bisco Inc. (Schaumburg, Illinois, USA)
Universal resin cement	Maxcem Elite Chroma	Kerr Corporation (Italy)
Adhesive system for dentin and enamel	Ambar	Dentscare LTDA (Brazil)

### Dentin samples preparation

**Figure 2:**
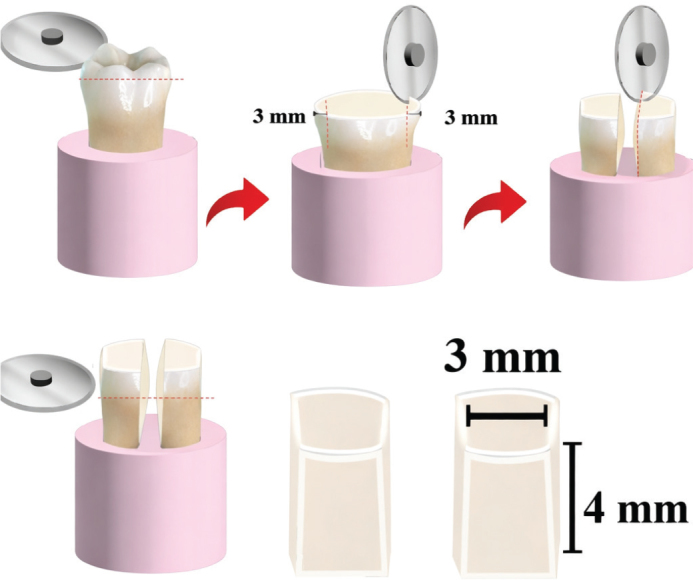
Schematic presentation of dentin specimen preparation.

Extracted permanent molars (*n* = 100) were collected and preserved in chloramine-T (0.5%) for 1 week followed by a thorough inspection under optical microscopy (Nikon Eclipse E 600, Nikon Corp.) at a magnification of 30×. Any stained or cracked teeth were not included. Flat rectangular specimens with exposed dentin were sectioned buccolingually (Isomet, Buehler), (Figure 2) [34] followed by polishing with #600 SiC sandpaper to obtain dentin samples with dimensions of 3×4×3 mm. Following preparation, the specimens underwent ultrasonic cleaning using 99% isopropanol for 5 min and then stored at 4°C in distilled water until further use.

### Surface treatments

**Figure 3:**
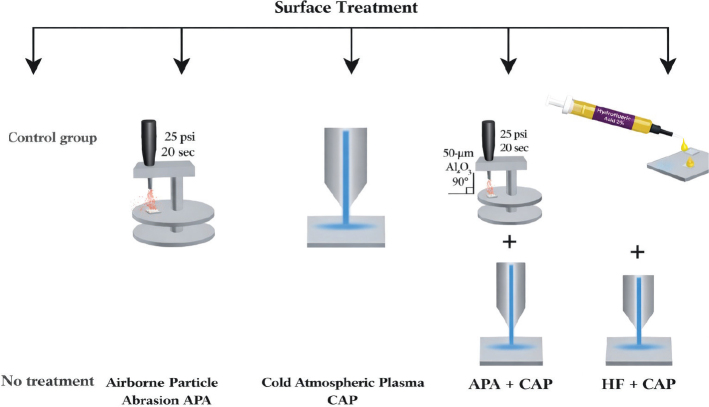
Schematic presentation of surface treatment of zirconia discs.

**Figure 4:**
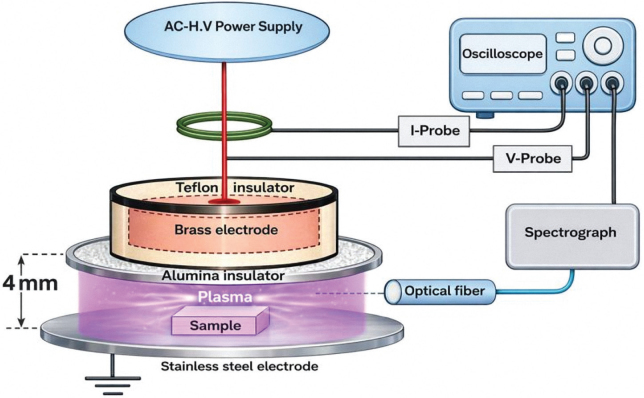
Diagram showing cold atmospheric plasma surface treatment.

The 200 specimens of 3Y-TZP and 5YSZ materials were subdivided into five subgroups each (*n* = 20) based on the surface treatments applied: (1) no surface treatment (Control), (2) APA [30], (3) CAP [35], (4) combination of APA and cold atmospheric plasma (APA+CAP), and (5) combination of 9.5% HF acid gel etching and CAP (HF+CAP) (Figures 3 and 4). The detailed surface treatment procedures are summarized in Table 2. The dentin surface was etched with phosphoric acid (37%) for 15s. Dentin bonding (Dentscare LTDA, Brazil) was then applied, left undisturbed for 20s, and light-cured (Elipar, 3M ESPE, Leicestershire, England) for 20s at an intensity of 1,350 mW/cm^2^. Zirconia primer (z-prime) was applied as a single layer to half of each cleaned and dried subgroup of 3Y-TZP (*n* = 50) and 5YSZ (*n* = 50) and left to dry.

**Table 2:** Zirconia surface treatment procedures.

Surface treatment	Description
Control group	Without any surface treatment
Airborne particle abrasion (APA)	Zirconia surfaces were air-abraded with 50 μm Al_2_O_3_ particles (Korox 50, BEGO, Germany) at 2 bar pressure, from a 10 mm distance, for 20 s, with the nozzle positioned perpendicular to the surface. A custom holder – comprising a wooden specimen base, a metal microblaster ring fixed with screws, and a connecting metal arm – was used to maintain perpendicular alignment with the specimen center [30].
Cold atmospheric plasma (CAP)	This group was treated with CAP prior to bonding using a dielectric barrier discharge air plasma system comprising two parallel metallic electrodes separated by a 4-mm gap. The upper brass electrode (45-mm diameter) was insulated with a Teflon sleeve, while the lower stainless-steel electrode (50-mm diameter, 3-mm thickness) was covered with a 2-mm alumina dielectric (80 × 80 mm). The upper electrode was powered by an AC high-voltage supply (Plasma Driver PVM500, Information Unlimited Co.) delivering a sinusoidal output up to 20 kV and 20 kHz.^35^
Combination of airborne particle abrasion and cold atmospheric plasma (APA+CAP)	Zirconia specimens subjected to APA and CAP surface treatment as mentioned above.
Combination of etching with 9.5% HF acid gel and cold atmospheric plasma (HF+CAP)	Etching of zirconia samples using 9.5% hydrofluoric acid gel (Porcelain Etch Gel, Bisco, USA) at ambient conditions for 1 h, followed by CAP surface treatment as mentioned above.

HF: hydrofluoric acid.

### Cementation procedures

Dentin was conditioned with phosphoric acid and a universal adhesive before using self-adhesive resin cement to standardize bonding and ensure consistent hybrid layer formation, following manufacturer guidelines and prior evidence of improved dentin adhesion.

Dentin and zirconia samples were cemented using the universal self-adhesive resin cement containing a clean-up indicator. The specimens were seated and sealed under a 5-kg constant load until cementation was completed [36]. Residual cement was gently removed with a microbrush, and the margins were light-cured (Elipar, 3M ESPE, Leicestershire, England) at 1,350 mW/cm^2^ for 40 s. The remaining specimens, 3Y-TZP (*n* = 50) and 5YSZ (*n* = 50), underwent the same procedure mentioned previously, excluding zirconia primer application. After adhesive cementation, all samples were placed in distilled water for 24 h at 37°C.

A thermocycler (SD Mechatronic, Thermocycler, Westerham, Germany) was used for thermal cycling of zirconia-dentin adhesive assemblies. The aging procedure included subjecting the adhesive assembly to 10,000 thermocycles between 5°C and 55°C, with a dwell time (30 s) and 10s transfer time [33].

### Shear bond strength

The zirconia/dentin specimens were tested for SBS using a universal mechanical tester (Instron) equipped with a beveled chisel rod. A load (5 kg) was applied with a crosshead speed of 0.5 mm/min. The force (*N*) needed to debond was recorded, and SBS was calculated according to the following equation: τ = F/A, where τ (shear bond strength (MPa)), F (force at failure), and A (bonding area; 3 × 4 = 12 mm^2^).

### Evaluation of failure modes

After testing SBS, failure modes were examined by optical microscopy (Nikon SMZ745T) at 30x and were classified as adhesive when > 90% of the debonded surface was at the interface, cohesive when fracture occurred within the substrate, and mixed when both adhesive and cohesive features were present. Two independent examiners evaluated all samples, resolving disagreements by discussion. Interexaminer agreement (Cohen’s kappa) was 0.87, indicating excellent reliability.

### Statistical analysis

SPSS version 20 (SPSS Inc.) was used for the statistical analysis. The normality of data distribution was assessed by Kolmogorov-Smirnov and Shapiro-Wilk tests. One-Way Analysis of Variance (ANOVA) and Bonferroni’s post hoc test were applied for the group and pairwise comparisons. Intragroup comparisons were performed using the independent t-test. A multiple-way ANOVA was utilized to investigate the interaction among study variables (surface treatment, type of zirconia, and with or without zirconia primer); *p*-values ≤ 0.05 were deemed statistically significant.

## Result

Surface treatment of zirconia showed statistically significantly higher SBS to dentin compared with the untreated surface (*P* < 0.001). Various zirconia materials revealed an insignificant effect on SBS; the bond strength of both materials was comparable. Combining APA and CAP surface treatment resulted in statistically significantly lower SBS than APA or CAP separate applications (*P* < 0.001). This is true for all variables, including different materials and with or without zirconia primer groups. Likewise, etching the zirconia surface in combination with CAP resulted in a statistically significantly lower SBS than with CAP alone (*P* < 0.001). The highest SBS values were observed in the APA and CAP groups when a zirconia primer is applied, irrespective of the zirconia material used. The lowest SBS values were recorded for the control untreated groups, in particular those cemented without primer application. The application of the zirconia primer resulted in a statistically significant enhancement in SBS, regardless of the type of zirconia materials or surface treatments employed (*P* < 0.001). Data comparing various groups are shown in Tables 3, 4, and supplementary Table 1.

**Table 3:** Shear bond strength (MPa) means and standard deviations across surface treatment groups.

Material	Surface treatment	Bonding protocol				Significance
		No primer		Primer		
		Mean	Std. Dev	Mean	Std. Dev
3Y-TZP	C	4.83 ^I^	0.85	7.56 ^H^	1.00	*p* < 0.001 and *p* > 0.05
	APA	17.01 ^E^	2.21	25.94 ^A^	3.10	
	CAP	17.43 ^E^	1.90	26.99 ^A^	1.71	
	APA+CAP	15.42 ^G^	0.93	24.40 ^B^	2.13	
	HF+CAP	15.14 ^G^	0.86	20.96 ^D^	1.32	
Significance		*p* < 0.001 and *p* > 0.05		*p* < 0.001 and *p* > 0.05		
5YSZ	C	4.49 ^I^	0.98	7.21 ^H^	0.76	*p* < 0.001 and *p* > 0.05
	APA	16.75^E^	1.74	26.27 ^A^	1.63	
	CAP	17.52 ^E^	1.26	26.89 ^A^	2.00	
	APA+CAP	16.47^F^	1.18	24.97 ^B^	1.47	
	HF+CAP	14.94 ^G^	1.23	22.26 ^C^	1.48	

CAP: cold atmospheric plasma; APA: airborne particle abrasion; HF: hydrofluoric acid; A to I : Different letters indicate statistically significant differences.

**Table 4:** Interaction of variables using the multiple-way ANOVA test for surface treatment, primer, and zirconia type.

Source	Type III sum of squares	Df	Mean square	*F*	*P*	Partial eta squared	Observed power
**Surface treatment (ttt)**	7129.33	4.00	1782.33	703.04	0.0001*	0.94	1.00
**Primer**	2697.01	1.00	2697.01	1063.84	0.0001*	0.86	1.00
**Zr (3Y & 5Y)**	2.19	1.00	2.19	0.86	0.354 ns	0.00	0.15
**Surface ttt × Primer**	319.18	4.00	79.79	31.47	0.0001*	0.41	1.00
**Surface ttt × Zr**	8.64	4.00	2.16	0.85	0.494 ns	0.02	0.27
**Primer × Zr**	0.96	1.00	0.96	0.38	0.539 ns	0.00	0.09
**Surface ttt × Primer × Zr**	6.22	4.00	1.55	0.61	0.654 ns	0.01	0.20

Based on the significance level *p* ≤ 0.05; ns: non-significant;

* significant.

**Figure 5:**
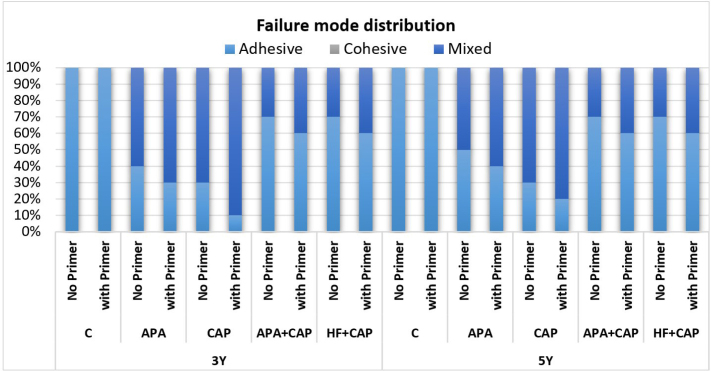
Stacked column chart of the distribution of failure modes of 3Y-TZP and 5YSZ.

**Figure 6:**
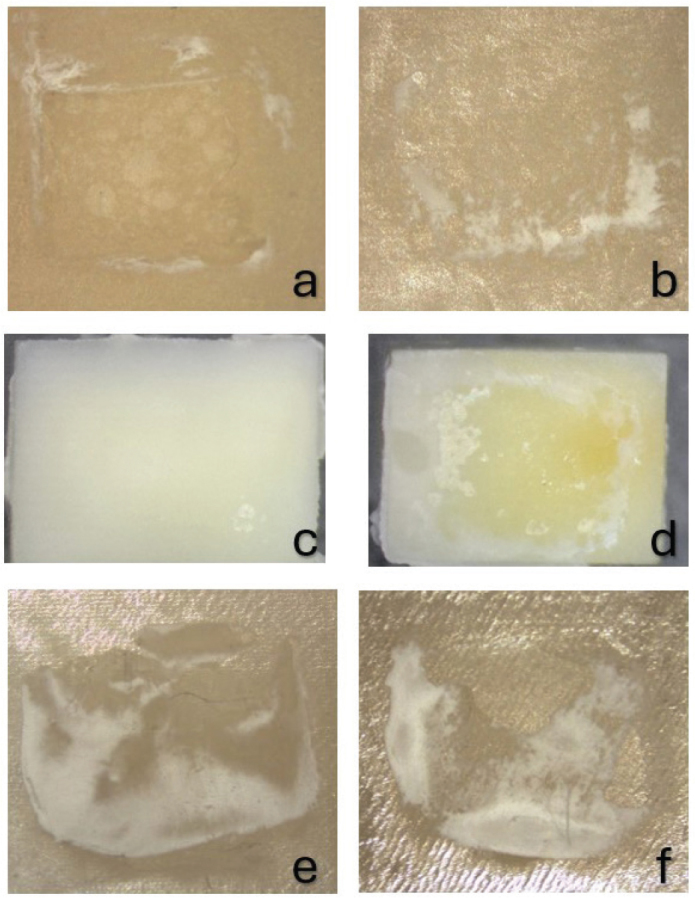
Optical microscopy images of four randomly selected specimens following the SBS test are presented; (a) and (b) exhibit complete failure at the interface, while (c) displays dentin specimen with the cement fully bonded to the dentin surface, indicating adhesive failure at the zirconia–cement interface. Image (d) demonstrates mixed failure, characterized by adhesive failure at the dentin–cement interface in the central region and at the zirconia–cement interface at the periphery. Image (e) reveals mixed failure, predominantly cohesive within the cement layer, while f illustrates mixed failure, with adhesive failure at both zirconia-cement and dentin–cement interfaces, as well as cohesive failure in the cement layer.

The modes of failure in the control groups without surface treatment were purely adhesive in all specimens, regardless of whether primer was applied. In contrast, all groups with zirconia surface treatments showed diverse modes of failure. APA and CAP exhibited adhesive and mixed failure modes, with a greater proportion of mixed failures observed. Conversely, the combination of APA and CAP, as well as CAP in conjunction with HF acid etching, demonstrated a higher prevalence of adhesive failures, as illustrated in Figures 5 and 6.

## Discussion

This laboratory study investigated the SBS of 3Y-TZP and 5YSZ to dentin under varying surface treatment protocols and with or without application of zirconia primer. The 1st null hypothesis that different surface treatments will not affect SBS was rejected, because our findings indicated that distinct surface treatments yielded varying levels of significance in SBS values. Conversely, the different zirconia materials exhibited comparable SBS values within similar groups; thus, the second null hypothesis was accepted. The third null hypothesis, asserting that zirconia primer application would not affect SBS, was rejected because the application significantly improved the SBS.

Many dental preparations often result in partial or complete dentin exposure, along with excessive loss of tooth structure that necessitates indirect restoration. Adhesion to dentin is a complex process and generally exhibits lower durability and reliability than bonding to enamel [9]. Moreover, APA has been the standard zirconia surface treatment for several decades, proven to be an effective method for preparing zirconia intaglio surfaces for adhesive bonding [11–14]. This technique is comparatively straightforward and cost effective. It is effective in producing surface roughness, increasing surface area for adhesion, and enhancing the wettability of the zirconia surface, thereby improving adhesion overall [14].

The findings of this study corroborate the established standard for surface treatment, demonstrating that APA of the zirconia surface resulted in significantly elevated SBS values compared with the combinations of APA and CAP or CAP plus HF. It is worth noting that the SBS values achieved with CAP treatment were comparable to, albeit slightly higher than, those obtained with APA, specifically when the zirconia primer was applied. The experimental design, particularly the APA component, aligns with the findings reported by Liu et al. [28]. which also noted a statistically significant difference in SBS among the APA treatment, primer application, and control conditions. Nevertheless, the SBS values recorded in the current study for APA treatment were significantly higher than those reported by Liu et al. [28]; this discrepancy may be attributed to the fact that Liu et al. subjected the zirconia-dentin cemented assembly to thermocycling and aging, represented by a 90-day water storage period. Furthermore, another study [37] investigated translucent zirconia surface treatment with cojet silica coating, which increased initial SBS. However, following aging, a dramatic decline in strength was observed, with SBS values recorded as 0 MPa for specimens without primers and only 3 MPa for those coated with primer post-silica treatment. This significant reduction in SBS may primarily stem from the use of the self-adhesive cement employed in that study, which utilized a custom-prepared experimental self-adhesive resin cement [37]. This phenomenon is further supported by an umbrella review of four systematic reviews, indicating that bonding to zirconia improves with surface roughening and 10-MDP-containing primers application [38].

Previous studies have indicated that CAP treatment has a nonsignificant effect on the surface roughness of zirconia [39, 40]. However, it was observed that CAP treatment resulted in a lower fluid contact angle on the zirconia surface than APA [39, 40]. Additionally, plasma surface treatment of zirconia has been shown to reduce carbon contamination [41] and enhance surface energy [39, 41], which may correlate with improved hydrophilicity and bonding potential of the zirconia surface. Given that CAP treatment does not involve surface impingement of roughening particles, the zirconia surface exhibits no changes in crystalline phases or microstructure [39, 41]. This suggests that CAP treatment does not induce premature phase transformations or the formation of microcracks, thus avoiding detrimental effects and leading to superior surface properties compared to those surfaces abraded with APA [40].

The findings of the present study are consistent with prior research, concluding that CAP is as effective as APA in terms of SBS [30, 39, 40, 42, 43].

HF etching alone has consistently demonstrated minimal or no enhancement in surface roughness or resin bonding to zirconia. However, recent research indicates that aggressive or prolonged HF etching may result in limited nanoscale surface modifications, albeit without yielding clinically significant improvements in bond strength [22]. In light of this context, the present study incorporated HF etching exclusively in conjunction with CAP to examine whether plasma-induced surface activation could amplify any minor physicochemical alterations induced by HF treatment [20, 28, 44]. Even when utilizing very high concentrations of HF acid, such as 40% [20, 44], with proper safety measures, including personal protective equipment and careful handling in a well-ventilated area, or when etching is prolonged for durations up to 320 min, these aggressive etching protocols may induce only minimal changes to zirconia surfaces at the nanoscale (i.e. within 120 nanometers), without resulting in any enhancement of bond strength [44]. These established protocols corroborate the findings of the present study, wherein HF etching demonstrated no significant effect on SBS values when used in conjunction with CAP. Furthermore, while both CAP and APA treatments improved SBS values of 3Y-TZP and 5YSZ materials, the combination of APA and CAP resulted in a lower bond strength compared to the application of either treatment individually. The roughness induced by APA can create uneven topography, which may hinder the effective interaction of CAP with the zirconia surface. This disruption could reduce the effective area for bonding and negatively impact the adhesion properties. Additionally, while the introduction of polar groups through plasma treatment is beneficial for enhancing bond strength, the roughened surface may lead to insufficient contact between the newly activated surface and the adhesive material. Indeed, the surface characteristics modified by APA may not favorably interact with the polar components introduced by CAP [45]. Comparison between 3Y-TZP and 5Y-SZ revealed no significant differences in SBS, indicating that zirconia material type did not influence bonding performance. Instead, bond strength was primarily determined by zirconia surface treatment and the use of appropriate adhesive protocols incorporating phosphate monomer–containing primers, particularly 10-MDP, irrespective of zirconia yttria content. This finding is supported by numerous studies reporting that 10-MDP is an essential component for achieving reliable and durable bonding to zirconia [11, 46–48].

In terms of the modes of failure, both 3Y-TZP and 5YSZ exhibited complete adhesive failure between zirconia and self-adhesive luting, suggesting a weak bond of untreated surfaces of zirconia [49, 50]. The groups subjected to APA or CAP surface treatments exhibited increased SBS, reflected in a failure mode characterized by mixed failure at zirconia–cement interface and cohesive failure in the cement layer. This finding underscores critical importance of surface treatment prior to adhesive procedures involving zirconia [13, 51], as well as the application of primer before cementation [52, 53].

This laboratory faced some limitations because only self-adhesive resin cement was utilized, and the dentin surface was treated exclusively with a universal adhesive without prior conditioning. The dentin-zirconia adhesive assembly was subjected solely to thermocycling, with no application of extensive aging or dynamic loading. Consequently, further research is recommended to incorporate more rigorous aging conditions to assess the durability of the bond between zirconia and dentin.

## Conclusions

Based on the findings of the present study, the following conclusions can be drawn:

1. Both CAP and APA produced comparable SBS; however, APA is recognized as the most straightforward and cost-effective alternative. The combination of CAP with APA or HF resulted in a slight decrease in SBS, which was deemed inefficient.
2. The application of a zirconia primer significantly enhanced SBS across all tested groups, underscoring its critical role in achieving reliable bonding between zirconia and dentin, irrespective of the surface treatment employed.
3. The type of zirconia material did not significantly influence SBS. Both 3Y-TZP and 5YSZ exhibited comparable and clinically acceptable bond strength to dentin when appropriate surface treatment and adhesive protocols were implemented.

## Supplementary Material



## Declarations

### Availability of data and material

The data will be made available as per requirement.
