# Supplementary material for: Role of cold atmospheric plasma alone or combined with conventional surface treatments on shear bond strength of 3Y-TZP and 5YSZ ceramics bonded to dentin
Source: Biomater Investig Dent. 2026 May 5;13:45563. doi: 10.2340/biid.v13.45563 (PMC13154724; doi:10.2340/biid.v13.45563)
Supplement: Supplementary file 1 [file BIiD-13-45563-s1.pdf]

Supplementary material has been published as submitted. It has not been copyedited or typeset by Biomaterial Investigations in Dentistry.

Supplementary Table 1: Descriptive statistics and comparison of shear bond strength between groups (ANOVA test)

Group		Mean	Std. Dev	95% Confidence Interval for Mean		Min	Max	P value
				Lower Bound	Upper Bound			
3Y-No Primer	C	4.83 [d]	.85	4.22	5.44	3.31	6.01	0.0001*
	APA	17.01 [a,b]	2.21	15.43	18.59	13.42	20.03	
	CAP	17.43 [a]	1.90	16.07	18.78	14.51	19.68	
	APA+CAP	15.42 [b,c]	.93	14.75	16.08	13.76	16.87	
	HF+CAP	15.14 [c]	.86	14.52	15.75	13.28	16.16	
3Y-With Primer	C	7.56 [p]	1.00	6.84	8.28	6.02	9.13	0.0001*
	APA	25.94 [m,n]	3.10	23.72	28.16	21.29	29.06	
	CAP	26.99 [m]	1.71	25.77	28.22	24.70	29.75	
	APA+CAP	24.40 [n]	2.13	22.87	25.92	20.57	27.32	
	HF+CAP	20.96 [o]	1.32	20.02	21.91	19.12	23.22	
5Y-No Primer	C	4.49 [t]	.98	3.79	5.19	3.06	5.72	0.0001*
	APA	16.75 [r]	1.74	15.51	18.00	14.78	20.00	
	CAP	17.52 [r]	1.26	16.62	18.42	15.16	18.68	
	APA+CAP	16.47 [r]	1.18	15.63	17.32	14.82	18.43	
	HF+CAP	14.94 [s]	1.23	14.06	15.81	13.44	16.96	
5Y-with primer	C	7.21 [z]	.76	6.67	7.75	6.16	8.29	0.0001*
	APA	26.27 [w,x]	1.63	25.10	27.43	24.49	29.13	
	CAP	26.89 [w]	2.00	25.45	28.32	23.78	29.57	
	APA+CAP	24.97 [x]	1.47	23.92	26.02	22.25	27.16	
	HF+CAP	22.26 [y]	1.48	21.20	23.32	20.12	24.41	

C: *Control,* ***APA:*** *Airborne particle abrasion,* ***CAP:*** *Cold plasma,* ***APA+CAP:*** *Cold plasma after Airborne particle abrasion,* ***HF+CAP:*** *Cold plasma with hydrofluoric acid.* ***3Y:*** *3 mol% yttria-partially stabilized monolithic zirconia.*
5Y: *5 mol% yttria-partially stabilized monolithic zirconia.*
Significance level p≤0.05, ** = Significant*
Post hoc test: *within the same comparison, means sharing the same superscript letters are not significantly different.*
